# Six-Month Follow-up of COVID-19 Patients: Mortality and Related Factors

**DOI:** 10.34172/aim.2022.89

**Published:** 2022-08-01

**Authors:** Mohammad Haji Aghajani, Mohammad Sistanizad, Amirmohammad Toloui, Arian Madani Neishaboori, Asma Pourhoseingholi, Ziba Asadpoordezaki, Reza Miri, Mahmoud Yousefifard

**Affiliations:** ^1^Prevention of Cardiovascular Disease Research Center, Shahid Beheshti University of Medical Sciences, Tehran, Iran; ^2^Department of Cardiology, School of Medicine, Shahid Beheshti University of Medical Sciences, Tehran, Iran; ^3^Department of Clinical Pharmacy, School of Pharmacy, Shahid Beheshti University of Medical Sciences, Tehran, Iran; ^4^Physiology Research Center, Iran University of Medical Sciences, Tehran, Iran; ^5^Department of Psychology, Maynooth University, Kildare, Ireland; ^6^Kathleen Lonsdale Institute for Human Health Research, Maynooth University, Kildare, Ireland; ^7^Pediatric Chronic Kidney Disease Research Center, Tehran University of Medical Sciences, Tehran, Iran

**Keywords:** COVID-19, Long-term follow up, Mortality, Risk factors

## Abstract

**Background:**

Currently, there is lack of evidence regarding the long-term follow-up of coronavirus disease 2019 (COVID-19) patients. The aim of this study is to present a 6-month follow-up of COVID-19 patients who were discharged from hospital after their recovery.

**Methods:**

This retrospective cohort study was performed to assess the six-month follow-up of COVID-19 patients who were discharged from the hospital between February 18 and July 20, 2020. The primary outcome was 6-month all-cause mortality.

**Results:**

Data related to 614 patients were included to this study. Of these 614 patients, 48 patients died (7.8%). The cause of death in 26 patients (54.2%) was the relapse of COVID-19. Also, 44.2% of deaths happened in the first week after discharge and 74.4% in the first month. Risk factors of all-cause mortality included increase in age (odds ratio [OR]=1.09; *P*<0.001), increase in neutrophil percentage (OR=1.05; *P*=0.009) and increase in heart rate (OR=1.06; *P*=0.002) on the first admission. However, the risk of all-cause death was lower in patients who had higher levels of hematocrit (OR=0.93; *P*=0.021), oxygen saturation (OR=0.90; *P*=0.001) and mean arterial pressure (OR=0.93; *P*=0.001). In addition, increase in age (OR=1.11; *P*<0.001) was an independent risk factor for COVID-19-related death, while higher levels of lymphocyte percentage (OR=0.96; *P*=0.048), mean arterial pressure (OR=0.93; *P*=0.006) and arterial oxygen saturation (OR=0.91; *P*=0.009) were protective factors against COVID-19-related deaths during the 6-month period after discharge.

**Conclusion:**

Death is relatively common in COVID-19 patients after their discharge from hospital. In light of our findings, we suggest that elderly patients who experience a decrease in their mean arterial pressure, oxygen saturation and lymphocyte count during their hospitalization, should be discharged cautiously. In addition, we recommend that one-month follow-up of discharged patients should be take place, and urgent return to hospital should be advised when the first signs of COVID-19 relapse are observed.

## Introduction

 Coronavirus disease 2019 (COVID-19), an emerging infectious disease, appeared in late 2019 and the number of infected people is still on the rise.^1^ In March 2020, the World Health Organization (WHO) declared a worldwide pandemic of COVID-19, because of the spread of the disease.^2,3^ The WHO has reported that the time from onset of the symptoms until death is between two to eight weeks.^4^ It is estimated that the mortality of this disease is about 3%, and the rate of mortality is directly related to age and underlying conditions.^5-7^ Also, in a study conducted on moderate to severe COVID-19 patients, the survival rate was estimated to be 87.7%.^8^

 Considering the pathogenicity of the severe acute respiratory syndrome coronavirus 2 (SARS-CoV-2) virus, various organs and systems in the body are affected in COVID-19, including the respiratory, cardiovascular, circulation, renal and even central nervous systems.^9-13^ These complications may decrease the patients’ quality of life and increase their early mortality after discharge from the hospital. In order to have accurate knowledge about the burden of COVID-19, long-term complications should be taken into account while calculating the mortality rate of the disease.^14-16^

 The mortality rate of COVID-19 has been reported to be 1–9% following discharge,^17-19^ but most of the studies have had a short follow-up period (maximum of two months). Because of the small number of studies with long-term follow-up periods, there is not enough evidence on this subject matter.

 As there is currently no comprehensive evidence for long-term follow-up of COVID-19 patients, the aim of the current study is to present a six-month follow-up of patients who were discharged from the hospital after their recovery. The main goal of this study is to assess the 6-month mortality rate and its related factors.

## Materials and Methods

###  Study Design and Setting

 The current retrospective cohort study was conducted in Imam Hossein Hospital, Tehran, Iran, on patients hospitalized from February 18 to July 20 2020. Patients’ data were recorded electronically in the hospital. Thus, the electronic database of the hospital was used to identify the patients hospitalized due to COVID-19, whose diagnosis was confirmed by real-time polymerase chain reaction (RT-PCR). Before collecting relevant data, the protocols of the study were approved by the ethics committee of Shahid Beheshti University of Medical Sciences. Researchers adhered to the Helsinki Declaration.

###  Participants

 In a consecutive sampling method, all hospitalized patients in Imam Hossein Hospital whose COVID-19 was confirmed by RT-PCR were enrolled in the present study. In-hospital death during the first admission, lack of data in patients’ profile and unresponsiveness of patients or their relatives after two phone calls during follow-up were considered as the exclusion criteria.

###  Data Collection and Variables

 In-hospital deaths during the first admission were identified and excluded. Next, a list of surviving patients who were discharged from the hospital was prepared. A checklist was designed and after studying the patients’ profiles, the researchers obtained the patients’ demographic characteristics, medical histories, vital signs at the time of admission, lab data, radiographic findings and date of hospital discharge. Data collection took place from February 18 to July 20, 2020 and follow-up of patients took place in November 2020. Thus, the follow-up time was six months at least and nine months at most.

 The extracted data included age, sex, body mass index (BMI), history of cardiovascular disorders, length of stay, incidence of cardiac complications during hospitalization, cardiac arrhythmia during hospitalization, mean value of vital signs during hospitalization, PvO_2_, PvCO_2_, red blood cell count, white blood cell count, percentage of lymphocyte and neutrophil, C-reactive protein, serum level of hemoglobin, creatinine, hematocrit, creatinine phosphokinase and COVID-19 treatment protocols.

 The hospital database was re-evaluated in November 2020 in order to identify the new hospitalized cases. Next, patient mortality was extracted from the records. In addition, a phone call was made to the patients, to ask about their mortality status. If the call was not answered, a second call was made 24 hours later. If other phone numbers were available on the record, those numbers were also tried. In order to reduce the missing data as much as possible, in cases of unresponsiveness, another phone call was made after one week. The outcome of the study was patient mortality, time of death and cause of death. Data was extracted from the patients’ files or obtained via phone calls.

###  Outcome

 The primary outcome of the present study was 6-month all-cause mortality. COVID-19-related death was assessed as secondary outcome.

###  Statistical Analysis

 Data were recorded and analyzed using the SPSS 22.0 statistical program. Quantitative data are reported as mean and standard deviation, or median and interquartile range, and qualitative data are reported as frequency and percentage.

 Patients were divided into two groups of alive and deceased. Then, using univariate analysis, the relationship between their demographics, clinical and laboratory findings in their first admission and their mortality after discharge were analyzed. Next, to adjust for confounding factors, a multivariate logistical regression with stepwise selection model was applied which included the backward approach with a significance level of less than 0.2. These models were fitted and in order to select the best variables for the multivariate model, the analysis was made in two separate sections. First, the relationship between possible risk factors and COVID-19-related mortality rate was assessed. Then, the relationship between these risk factors with all-cause mortality was explored. In all of the analyses, p values less than 0.05 were considered significant.

 In the longest follow-up reported in previous studies (which was 2 months), the prevalence of mortality of COVID-19 patients after discharge has been reported to be 9.1%.^17^ Therefore, considering a 95% confidence interval and an error of 2.5%, the minimum sample size required for the current study was calculated to be 509 patients.

## Results

 Six-month follow-up of COVID-19 patients showed that of the 734 patients discharged from the hospital, 120 patients (16.34%) did not answer the phone calls. Therefore, ultimately 614 patients were enrolled in the current study (Figure 1). Comparison of baseline characteristics of included patients and excluded patients in follow-up was reported in Table S1 (Supplementary file 1). From these 614 patients, 48 passed away (7.8%) during the follow-up period. The cause of death was relapse of COVID-19 in 26 patients (54.2%), cancer-related death in 6 patients (12.5%) and myocardial infarction/stroke in 3 patients (6.8%). One patient (2.1%) passed away due to drug abuse (drug overdose) and the cause of death was unknown for 12 patients (25.0%) (Figure 2). In the assessment of time of death, it was found that 44.2% of the deaths after discharge occurred in the first week, and 74.4% of them took place within the first month after discharge (Figure 2).

**Figure 1:**
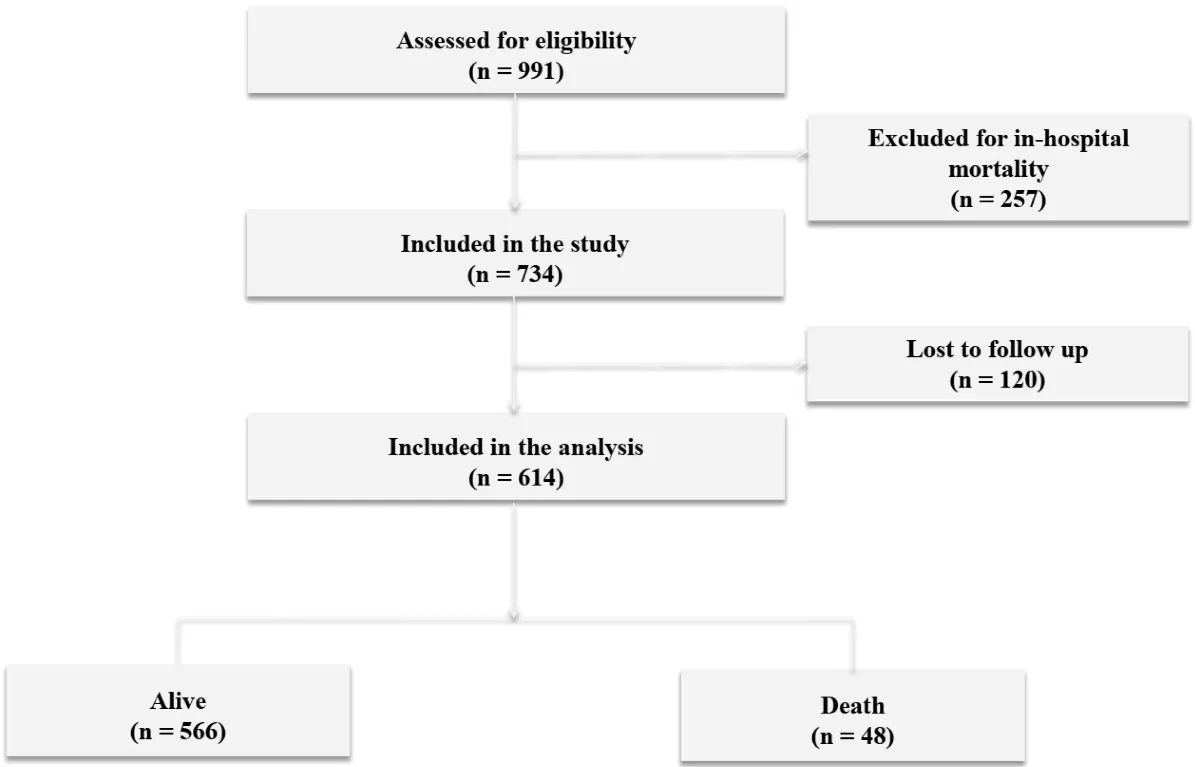
Flow Diagram of the Study

**Figure 2:**
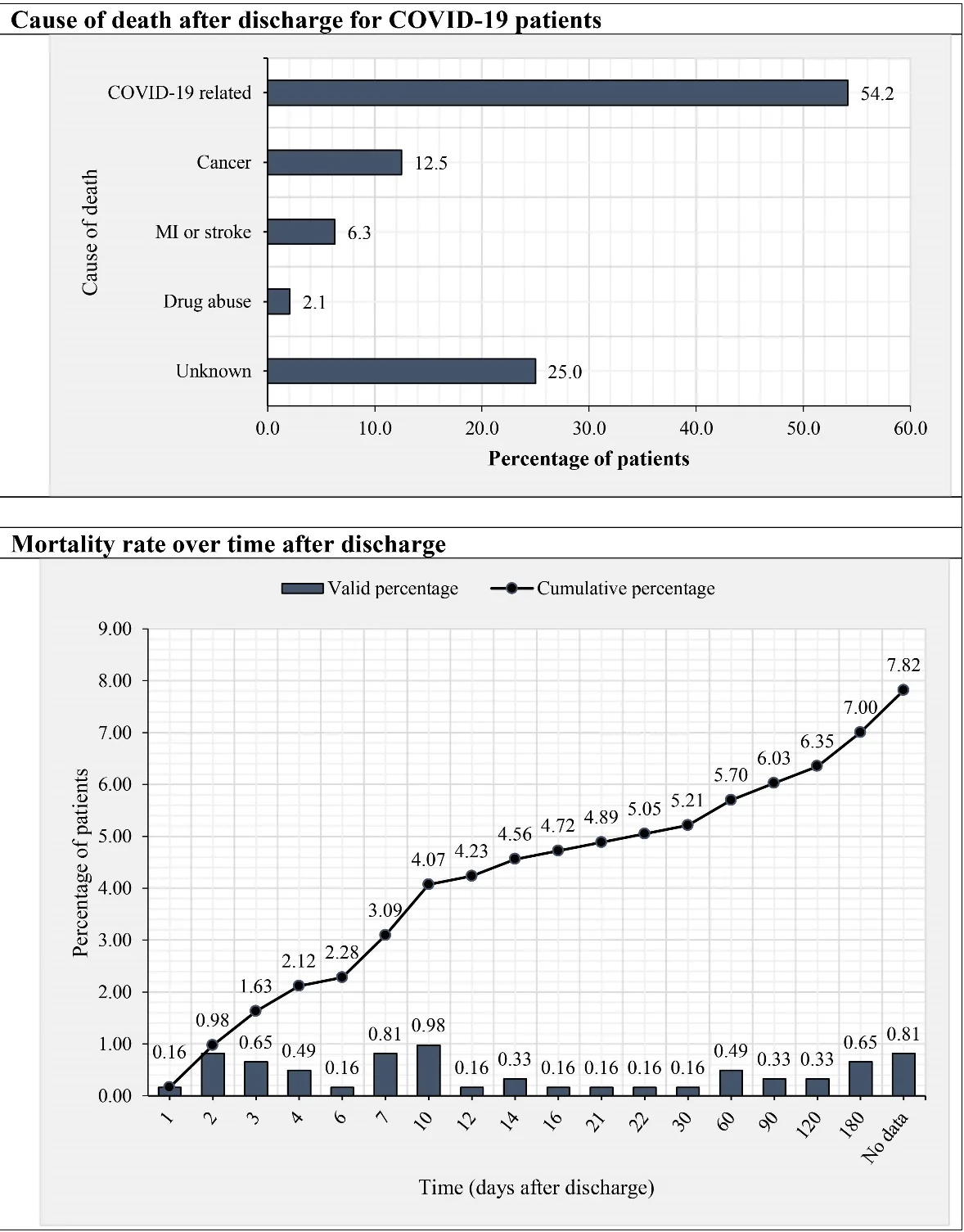
Cause of Death and Mortality Rate Over Time in COVID-19 Patients after Discharge

 The mean age of alive and deceased patients was 57.4 ± 16.1 years, and 73.7 ± 15.4 years, respectively (*P* < 0.001). BMI of the deceased group was slightly lower than that of the alive group (25.1 vs 26.5; *P*< 0.001). In addition, in the deceased patients, history of cardiovascular diseases (43.8% vs 27.4%; *P* = 0.016) was significantly higher than that of the alive group. White blood cells count (9.0 vs 6.5 count/mm^3^; *P* < 0.001), neutrophil percentage (72.8% vs 77.6%; *P*= 0.001) and serum creatinine levels (1.3 vs 1.1 mg/dL; *P*= 0.004) were significantly higher in the deceased group. On the contrary, SpO_2_ levels (89.3% vs 92.0%; *P*< 0.001), mean arterial pressure (82.2 vs 86.6 mmHg; *P*= 0.013), lymphocyte percentage (14.6% vs 20.0%; *P*< 0.001), hemoglobin levels (12.0 vs 12.6 mg/dL; *P*= 0.014), and red blood cell count (4.2 vs 4.5 count/mm^3^; *P*= 0.015) were lower in the deceased group. Different COVID-19 treatment protocols in the first admission did not have an impact on the six-month mortality rate of the patients (*P*= 0.286) (Table 1).

**Table 1:** Distribution of Baseline and Hospitalization-Related Variables According to Patient Outcomes

**Variable**	**Alive (n=566)**	**Dead (n=48)**	**Total (N=614)**	**Odds Ratio (95% CI)**	***P***
Age (y)	57.4 ± 16.1	73.7 ± 15.4	58.6 ± 16.7	1.08 (1.05, 1.10)	< 0.001
Sex (%)					
Women	274 (48.8)	24 (50.0)	298 (48.5)	*Reference*	
Men	292 (51.2)	24 (50.0)	316 (51.5)	0.94 (0.52, 1.69)	0.832
Body mass index (kg/m^2^)	26.5(24.2–29.5)	25.1(22.5–26.9)	26.3 (24.2-29.4)	0.88 (0.81, 0.95)	0.002
History of cardiovascular disorders (%)					
No	411 (72.6)	27 (56.3)	438 (71.3)	*Reference*	
Yes	155 (27.4)	21 (43.8)	176 (28.7)	2.06 (1.12, 3.76)	0.018
Length of stay (days)	6 (4-9)	5 (3-8)	6 (4-9)	0.96 (0.90, 1.03)	0.219
Incidence of cardiac complication during hospitalization (%)					
No	530 (93.6)	42 (87.5)	572(93.2)	*Reference*	
Yes	36(6.4)	6 (12.5)	42(6.8)	2.10 (0.84, 5.28)	0.113
Cardiac arrhythmia during hospitalization (%)					
No	266 (52.4)	22 (48.9)	340 (51.4)	*Reference*	
Yes	242 (47.6)	23 (51.1)	321 (48.6)	1.15 (0.62, 2.12)	0.655
Vital signs during hospitalization					
Mean temperature (°C)	37.0 (36.8–37.3)	37.0 (36.9–37.3)	37.0 (36.8–37.3)	0.997 (0.98, 1.14)	0.964
Mean heart rate (per minute)	83.3 (79.3–88.6)	85.0 (81.2–90.2)	83.3 (79.7–89.0)	1.02 (0.99, 1.05)	0.147
Mean oxygen saturation (%)	92.0 (90.0–94.0)	89.3 (86.3–92.3)	91.7 (89.7–94.0)	0.90 (0.85, 0.95)	< 0.001
Mean respiratory rate (per minute)	18.7 (17.7–21.3)	19.0 (17.2–22.2)	18.7 (17.7–21.6)	1.00 (94, 1.06)	0.940
Mean of Mean arterial pressure (mm Hg)	86.6 (81.1–91.1)	82.2(77.2–90)	86.6(81.1–91.1)	0.95 (0.91, 0.99)	0.011
Laboratory assessment					
PvO_2_ (mm Hg)	29.2 (23.2–39.6)	29.9 (23.1–47.2)	29.2 (23.2–40.0)	1.01 (0.99, 1.02)	0.442
PvCO_2_ (mm Hg)	42.1 (37.5–46.3)	42.2 (35.6–46.7)	42.1 (37.5–46.3)	1.01 (0.98, 1.05)	0.547
White blood cell count (per mm^3^)	6.5 (4.9–8.5)	9.0 (6.5–11.7)	6.6 (5.0–8.8)	1.04 (1.01, 1.07)	0.026
Lymphocyte (%)	20.0 (13.2–27.0)	14.6 (8.0–21.9)	20.0 (12.9–26.6)	0.94 (0.90, 0.97)	0.001
Neutrophil (%)	72.8 (65.5–80.0)	77.6 (70.6–87.3)	73.2 (65.6–80.3)	1.06 (1.03, 1.10)	< 0.001
C-reactive protein (mg/L)	44.5 (18.1–71.8)	46.5 (33.0–83.0)	44.6 (19.1–72.0)	1.00 (0.996, 1.008)	0.563
Hemoglobin (mg/dL)	12.6 (11.5–13.7)	12.0 (10.2–13.2)	12.6 (11.4–13.7)	0.81 (0.70, 0.94)	0.005
Red blood cell count (per mm^3^)	4.5 (4.1–4.9)	4.2 (3.8–4.7)	4.4(4.1–4.8)	0.55 (0.36, 0.86)	0.008
Creatinine (mg/dL)	1.1 (1.0–1.4)	1.3 (1.0–1.9)	1.2 (1.0–1.4)	1.08 (0.89, 1.30)	0.445
Hematocrit (%)	37.5 (34.4–40.6)	36.0 (31.4–39.5)	37.5(34.2–40.5)	0.94 (0.89, 0.98)	0.011
Creatinine phosphokinase (mcg/L)	105.0 (58–223)	85.5 (55.8–196.0)	103.0 (58.0–222.0)	1.00 (0.999, 1.001)	0.420
Treatment protocols*					
Corticosteroid + interferon + remdesivir	11 (1.9)	0 (0.00%)	11(1.8)	---	0.286
Corticosteroid + interferon + favipiravir	13 (2.3)	0 (0.00%)	13(2.1)	---	
Corticosteroid + interferon + Kaletra + hydroxychloroquine	49 (8.7)	5 (10.4)	54 (8.8)	---	
Corticosteroid + interferon + Kaletra	31 (5.5)	1 (2.1)	32(5.2)	---	
Interferon + Kaletra + hydroxychloroquine	42(7.4)	5 (10.4)	47 (7.7)	---	
Interferon + Kaletra	25 (4.4)	5 (10.4)	30 (4.9)	---	
Kaletra + hydroxychloroquine	86(15.2)	6 (12.5)	92 (15.0)	---	
Hydroxychloroquine	166 (29.3)	9 (18.80)	175 (28.5)	---	
Others	143 (25.3)	17 (35.40)	160 (26.1)	---	

CI, Confidence interval; PvCO_2_, Venous carbon dioxide tension; PvO_2_, venous oxygen tension. *P value was reported based on chi-square test since the logistic model was not fitted.

 Multivariate analyses showed that independent risk factors of all-cause mortality including increase in age (OR = 1.09; 95% CI: 1.06, 1.13; *P*< 0.001), increase in neutrophil percentage (OR = 1.05; 95% CI: 1.01, 1.09; *P*= 0.009) and increase in heart rate (OR = 1.06; 95% CI: 1.02 1.10; *P*= 0.002), in the first admission, were significantly related to the mortality rate after discharge. On the other hand, the risk of all-cause death was lower in patients who had higher levels of hematocrit (OR = 0.93; 95% CI: 0.87, 0.99; *P* = 0.021), oxygen saturation (OR = 0.90; 95% CI: 0.84, 0.95; *P* = 0.001) and mean arterial pressure (OR = 0.93; 95% CI: 0.89, 0.97; *P*= 0.001) (Table 2).

**Table 2:** Multivariate Regression for Prediction of 6-Month Morality of COVID-19 Patients

**Variable**	**Odds Ratio**	**95% Confidence Interval**	***P***
COVID-19 related mortality			
Age	1.11	1.07 – 1.15	< 0.001
Lymphocyte percentage	0.96	0.91 – 1.00	0.048
Mean arterial pressure	0.93	0.88 – 0.98	0.006
Mean oxygen saturation	0.91	0.85 – 0.98	0.009
All-cause mortality			
Age	1.09	1.06 – 1.13	< 0.001
Duration of hospitalization	0.94	0.86 – 1.01	0.098
Hematocrit	0.93	0.87 – 0.99	0.021
Neutrophil percentage	1.05	1.01 – 1.09	0.009
Mean oxygen saturation	0.90	0.84 – 0.95	0.001
Mean arterial pressure	0.93	0.89 – 0.97	0.001
Heart rate	1.06	1.02 – 1.10	0.002

SpO_2_, Oxygen saturation.

 In addition, the analyses showed that increase in age (OR = 1.11; 95% CI: 1.07, 1.15; *P*< 0.001) was an independent risk factor for COVID-19-related death. However, increase in lymphocyte percentage (OR = 0.96; 95% CI: 0.91, 1.00; *P*= 0.048), increase in mean arterial pressure (OR = 0.93; 95% CI: 0.88, 0.98; *P* = 0.006) and increase in oxygen saturation (OR = 0.91; 95% CI: 0.85, 0.98; *P* = 0.009) were the independent protective factors against COVID-19-related mortality in the first six months following hospital discharge.

## Discussion

 The findings of present study showed that the six-month mortality rate of COVID-19 patients, who recovered and were discharged from the hospital, was 7.8%. Of these deaths, 54.2% were directly related to COVID-19 and 20.8% of the deaths happened due to cardiac complications and stroke. Considering the high prevalence of cardiac complications and stroke in COVID-19 patients,^10,13^ it is also possible that these deaths are indirectly related to COVID-19, as well. Thus, it seems that 75% of the deaths in the first six months after recovery are related to COVID-19 or its long-term complications.

 The incidence of cardiovascular complications in COVID-19 patients has been shown in previous studies and the findings suggest that the incidence of these complications is independent from the past medical history of patients’ cardiac disease.^20^ These findings are in line with the idea that cardiovascular deaths observed in COVID-19 patients are a result of the direct effects of the disease on the cardiovascular system, rather than past medical history of the patients. This fact was also shown in the current study, because no relationship was found between the past medical history of cardiovascular diseases and the six-month mortality rate of the patients.

 In line with previous studies, which mostly reported older age as a risk factor for in-hospital mortality,^6,7,21,22^ we concluded that age is also an independent risk factor for out-of-hospital mortality after discharge. This is an alarm for health care providers and policy makers to put in place an organized follow-up for older COVID-19 patients after their discharge from the hospital.

 In the current study, levels of oxygen saturation in the first admission were shown to be significantly related to the six-month mortality rate. These low levels of oxygen saturation are in direct relationship with the levels of lung involvement; therefore, the significant relationship between lower levels of oxygen saturation and mortality rate is expected.

 Mean arterial pressure has a direct relationship with cardiac output and systemic vascular resistance. In the current study, the relationship between the decrease in mean arterial pressure during hospitalization and six-month mortality may be due to the decrease in cardiac output as a result of direct damage caused by the SARS-CoV-2 virus to cardiac tissue. Since, COVID-19-related dysautonomia of vasculature has been reported in previous studies, the effects of the disease on vascular wall should not be dismissed.^23^ Therefore, the decrease in mean arterial pressure and its relation to the 6-month mortality of patients can be attributed to the decrease in cardiac output and dysautonomia of vasculature in COVID-19 patients. Although studies show that history of hypertension is related to higher mortality in COVID-19 patients, recent findings suggest that low blood pressure in the course of the disease can also elevated the risk of mortality in these patients. For example, a study showed that using ACE inhibitors/ARB drugs in the time of admission in patients, is related to higher rates of in-hospital deaths.^24^ Also, a study on 6493 COVID-19 patients showed that hypotension has a direct relationship with higher rates of in-hospital deaths.^25^

 One of the interesting findings of the current study is the 44.2% incidence of deaths during the first week, and the 74.4% incidence of death in the first month after discharge. Therefore, it might be possible to reduce the number of deaths by designing an at least one-month follow-up protocol for the patients. In comparison to our study, Somani et al showed that in 14-day follow-up of discharged patients, the mortality rate was 3.4%,^19^ whereas the mortality in the same time period was 7.8% in our study. The cause of this difference might be related to the inclusion of only re-admitted patients to the first admitted hospital, while in our study not only were the readmitted patients evaluated, but also all patients were followed using phone calls and deaths which happened at home or other hospitals were also recorded. In another study, with a follow-up period of 60 days, Donnelly et al. reported that 9.1% of the discharged patients passed away in 2 months.^17^ This number is closer to the data reported in the current study. The reported mortality rate by Donnelly et al is slightly higher than that in our study, which may be because the evaluated patients were older in their study compared to ours.

 In a study conducted by Yeo et al, the 30-day mortality rate of discharged patients was reported to be 1% of the whole population, which is much lower than other studies.^18^ The most important cause of this difference is the method of follow-up used in their study, where only patients who had been re-admitted to the first admitted hospital were included.

 One of the strengths of the current study is that all discharged patients were followed, while in other similar studies, only the mortality rate of readmitted patients to the first hospital was analyzed. This may lead to an underestimation in the mortality rate after discharge, because some of the patients are readmitted to other medical centers or pass away at home. In order to prevent this bias, in addition to re-evaluation of readmitted patient profiles in Imam Hossein Hospital, a phone call was made to patients or their next of kin in order to estimate a more exact mortality rate for COVID-19 patients after discharge. Still, the attrition of 120 COVID-19 patients after discharge, in spite of multiple phone calls, is a limitation of the current study. Even though this 16% missing data is a limitation, in cohort studies, a cut-off point of 20% is considered to be the threshold which can cause serious bias in findings.^26^ Also, in 25% of the patients, the cause of death was unknown.

 In conclusion, death is a relatively common consequence of COVID-19 after recovery and discharge from the hospital. The findings of the current study suggest that the six-month mortality rate of discharged COVID-19 patients is 7.8%. These deaths are related to increase in age and decrease in mean arterial pressure, oxygen saturation and lymphocyte percentage during the first hospitalization. Thus, we suggest that the older patients who experience a decrease in mean arterial pressure, decrease in oxygen saturation or a decrease in lymphocyte count during their hospitalization, should be discharged cautiously. Also, our findings show a 74.4% mortality rate after 1st month of discharge. Therefore, we strongly recommend that one-month follow-up of discharged patients should take place, and urgent return to the hospital should be advised with the first signs of relapse of COVID-19.

## Supplementary Materials

Supplementary file 1 contains Table S1.

## References

[1] Wang C, Horby PW, Hayden FG, Gao GF (2020). A novel coronavirus outbreak of global health concern. Lancet.

[2] World Health O. Coronavirus disease 2019 (‎COVID-19)‎: situation report, 92 Geneva: World Health Organization; 2020 [updated 2020-04- 21; Available from: https://www.who.int/docs/default-source/coronaviruse/situation-reports/20200421-sitrep-92-covid-19.pdf.

[3] World Health Organization. Coronavirus disease (‎ COVID-19)‎ Genova: World Health Organization; 2020. Available from: https://www.who.int/emergencies/diseases/novel-coronavirus-2019.

[4] World Health Organization. Report of the WHO-China joint mission on coronavirus disease 2019 (COVID-19) Geneva: World Health Organization; 2020. Available from: https://www.who.int/docs/default-source/coronaviruse/who-china-joint-mission-on-covid-19-final-report.pdf.

[5] Emami A, Javanmardi F, Pirbonyeh N, Akbari A (2020). Prevalence of Underlying Diseases in Hospitalized Patients with COVID-19: a Systematic Review and Meta-Analysis. Arch Acad Emerg Med.

[6] Khateri S, Mohammadi H, Khateri R, Moradi Y (2020). The Prevalence of Underlying Diseases and Comorbidities in COVID-19 Patients; an Updated Systematic Review and Meta-analysis. Arch Acad Emerg Med.

[7] Zali A, Gholamzadeh S, Mohammadi G, Looha MA, Akrami F, Zarean E (2020). Baseline Characteristics and Associated Factors of Mortality in COVID-19 Patients; an Analysis of 16000 Cases in Tehran, Iran. Arch Acad Emerg Med.

[8] Sousa G, Garces T, Cestari V, Florêncio R, Moreira T, Pereira M (2020). Mortality and survival of COVID-19. Epidemiol Infect.

[9] Darabi P, Kalo MB, Ali KM, Safari S, Yousefifard M, Hosseini M (2020). COVID-19: Features, clinical course and concerns. J Cell Mol Anesth.

[10] MadaniNeishaboori A, Moshrefiaraghi D, Ali KM, Toloui A, Yousefifard M, Hosseini M (2020). Central Nervous System Complications in COVID-19 Patients; a Systematic Review and Meta-Analysis based on Current Evidence. Arch Acad Emerg Med.

[11] Safari S, Mehrani M, Yousefifard M (2020). Pulmonary thromboembolism as a potential cause of clinical deterioration in COVID-19 patients; a commentary. Arch Acad Emerg Med.

[12] Safari S, Yousefifard M (2021). A guideline for the prevention and treatment of thromboembolism in COVID-19 patients. J Emerg Pract Trauma.

[13] Toloui A, Moshrefiaraghi D, Madani Neishaboori A, Yousefifard M, Haji Aghajani M (2021). Cardiac Complications and Pertaining Mortality Rate in COVID-19 Patients; a Systematic Review and Meta-Analysis. Arch Acad Emerg Med.

[14] Carfì A, Bernabei R, Landi F (2020). Persistent symptoms in patients after acute COVID-19. JAMA.

[15] Puntmann VO, Carerj ML, Wieters I, Fahim M, Arendt C, Hoffmann J (2020). Outcomes of cardiovascular magnetic resonance imaging in patients recently recovered from coronavirus disease 2019 (COVID-19). JAMA Cardiol.

[16] SeyedAlinaghi S, Afsahi AM, MohsseniPour M, Behnezhad F, Salehi MA, Barzegary A (2021). Late Complications of COVID- 19; a Systematic Review of Current Evidence. Arch Acad Emerg Med.

[17] Donnelly JP, Wang XQ, Iwashyna TJ, Prescott HC (2021). Readmission and death after initial hospital discharge among patients with COVID-19 in a large multihospital system. JAMA.

[18] Yeo I, Baek S, Kim J, Elshakh H, Voronina A, Lou MS (2021). Assessment of Thirty‐Day Readmission Rate, Timing, Causes, and Predictors after Hospitalization with COVID‐19. J Intern Med.

[19] Somani SS, Richter F, Fuster V, De Freitas JK, Naik N, Sigel K (2020). Characterization of patients who return to hospital following discharge from hospitalization for COVID-19. J Gen Intern Med.

[20] Becker RC (2020). Anticipating the long-term cardiovascular effects of COVID-19. J Thromb Thrombolysis.

[21] Guan WJ, Liang WH, Zhao Y, Liang HR, Chen ZS, Li YM, et al. Comorbidity and its impact on 1590 patients with COVID-19 in China: a nationwide analysis. Eur Respir J 2020;55(5). 10.1183/13993003.00547-2020. PMC7098485 32217650

[22] Zhou F, Yu T, Du R, Fan G, Liu Y, Liu Z (2020). Clinical course and risk factors for mortality of adult inpatients with COVID-19 in Wuhan, China: a retrospective cohort study. Lancet.

[23] Pennisi M, Lanza G, Falzone L, Fisicaro F, Ferri R, Bella R (2020). SARS-CoV-2 and the nervous system: from clinical features to molecular mechanisms. Int J Mol Sci.

[24] Selçuk M, Çınar T, Keskin M, Çiçek V, Kılıç Ş, Kenan B (2020). Is the use of ACE inb/ARBs associated with higher in-hospital mortality in Covid-19 pneumonia patients?. Clin Exp Hypertens.

[25] Mikami T, Miyashita H, Yamada T, Harrington M, Steinberg D, Dunn A (2021). Risk factors for mortality in patients with COVID-19 in New York City. J Gen Intern Med.

[26] National Institutes of Health. Study Quality Assessment Tools| National Heart, Lung, and Blood Institute (NHLBI): Medline; 2014. Available from: https://www.nhlbi.nih.gov/health-topics/study-quality-assessment-tools.

