# Supplementary material for: Six-Month Follow-up of COVID-19 Patients: Mortality and Related Factors
Source: Arch Iran Med. 2022 Aug 1;25(8):557–63. doi: 10.34172/aim.2022.89 (PMC13661080; doi:10.34172/aim.2022.89)
Supplement: Supplementary file 1 [file aim-25-557-s001.pdf]

Arch Iran Med. August 2022;25(8):557-563
10.34172/aim.2022.89

Six-Month Follow-up of COVID-19 Patients: Mortality and Related Factors

Mohammad Haji Aghajani, MD[1,2]; Mohammad Sistanizad[1,3]; Amirmohammad Toloui[4]; Arian Madani Neishaboori[4]; Asma Pourhoseingholi, PhD[1]; Ziba Asadpoordezaki, PhD Student[5,6]; Reza Miri, MD[1,2*]; Mahmoud Yousefifard PhD[4,7*]

[1]Prevention of Cardiovascular Disease Research Center, Shahid Beheshti University of Medical Sciences, Tehran, Iran
[2]Department of Cardiology, School of Medicine, Shahid Beheshti University of Medical Sciences, Tehran, Iran
[3]Department of Clinical Pharmacy, School of Pharmacy, Shahid Beheshti University of Medical Sciences, Tehran, Iran
[4]Physiology Research Center, Iran University of Medical Sciences, Tehran, Iran
[5]Department of Psychology, Maynooth University, Kildare, Ireland
[6]Kathleen Lonsdale Institute for Human Health Research, Maynooth University, Kildare, Ireland
[7]Pediatric Chronic Kidney Disease Research Center, Tehran University of Medical Sciences, Tehran, Iran

Supplementary file 1

Table S1: Comparison of baseline characteristics of included patients and excluded patients in follow-up.

Variable	Follow up group N= 614	Lost to follow up group N=120	Total N=734	P
**Age (year)**	58.6±16.1	57.2±17.3	58.6±16.7	0.379
**Sex (%)**				
Women	298 (48.5)	49 (40.8)	347 (47.3)	0.122
Men	316 (51.5)	71 (59.2)	387 (52.7)	
**Body mass index (kg/m^2)**	27.2±4.9	27±4.8	27.2±4.9	0.664
**History of cardiovascular disorders (%)**				
No	438 (71.3)	89 (74.2)	527 (71.8)	0.528
Yes	176 (28.7)	31 (25.8)	207 (28.2)	
**Length of stay (days)**	7.4±5.5	8.2±6.4	7.5±5.6	0.274
**Incidence of cardiac complication during hospitalization (%)**				
No	572 (93.2)	115 (95.8)	687 (93.6)	0.274
Yes	42 (6.8)	5 (4.2)	47 (6.4)	
**Cardiac arrhythmia during hospitalization (%)**				
No	64 (53.3)	349 (56.8)	413 (56.3)	0.479
Yes	56 (46.7)	265 (43.2)	321 (43.7)	
**Vital sign during hospitalization**				
Mean temperature (Celsius)	37 (36.8-37.2)	37.1 (36.9-37.4)	37 (36.8-37.2)	0.962

Mean heart rate (per minute)	83 (79.3-89)	85.3 (80-91)	83.3 (79.6-89)	0.122
Mean oxygen saturation (%)	91.6 (89.6-94)	92.3 (90-94.6)	91.6 (89.6-94)	0.059
Mean respiratory rate (per minute)	18.6 (17.6-21.5)	19 (17.6-23.3)	18.6 (17.6-21.6)	0.204
Mean of Mean arterial pressure (mmHg)	86.6 (81.1-91.1)	86.6 (80-90)	86.6 (81.1-91.1)	0.415
**Laboratory assessment**				
PvO_2 (mmHg)	29.2 (23.2-40)	30.6 (23.9-39)	29.3 (23.3-39.9)	0.671
$PvCO_2$ (mmHg)	42.1 (37.5-46.3)	43.3 (36.7-47.7)	42.2 (37.4-46.6)	0.604
White blood cell count (per mm^3)	6.6 (5-8.8)	6.5 (5.1-8.7)	6.6 (5-8.7)	0.997
Lymphocyte (%)	20 (12.9-26.7)	20 (13.1-25.4)	20 (13-26)	0.925
Neutrophil (%)	73.21 (65.6-80.3)	74 (67-80.8)	73.5 (65.8-80)	0.484
C-reactive protein (mg/L)	44.6 (19.1-72)	37.3 (20.5-65.2)	44 (19.6-71.2)	0.307
Hemoglobin (mg/dL)	12.6 (11.4-13.7)	13.1 (11.7-14.2)	12.6 (11.5-13.8)	0.077
Red blood cell count (per mm^3)	4.4 (4-4.8)	4.5 (4.2-4.9)	4.4 (4-4.8)	0.076
Creatinine (mg/dL)	1.2 (1-1.4)	1.1 (0.9-1.4)	1.2 (1-1.4)	0.342
Hematocrit (%)	37.5 (34.2-40.5)	39 (35.2-41.9)	37.6 (34.3-40.7)	0.044
Creatinine phosphokinase (mcg/L)	103 (58-222)	108.5 (58.7-286.7)	103 (58-226.5)	0.348
**Treatment protocols**				
Corticosteroid + interferon + remdesivir	11 (1.8)	2 (1.7)	13 (1.8)	
Corticosteroid + interferon + favipiravir	12 (2.1)	1 (0.8)	14 (1.9)	
Corticosteroid + interferon + kaletra + hydroxychloroquine	54 (8.8)	10 (8.3)	64 (8.7)	0.722
Corticosteroid + interferon + kaletra	32 (5.2)	4 (3.3)	36 (4.9)	
interferon + kaletra + hydroxychloroquine	47 (7.7)	8 (6.7)	55 (7.5)	
Interferon + kaletra	30 (4.9)	5 (4.2)	35 (4.8)	
Kaletra + hydroxychloroquine	92 (15)	25 (20.8)	117 (15.9)	
Hydroxychloroquine	175 (28.5)	39 (32.5)	214 (29.2)	
Others	160 (26.1)	26 (21.7)	186 (25.3)	

$PvCO_2$: Venous carbon dioxide tension; PvO_2: venous oxygen tension
